# Bacterial immune systems as causes and consequences of microbiome structure

**DOI:** 10.1371/journal.pbio.3003489

**Published:** 2025-11-19

**Authors:** Rafael Custodio, Ellinor O. Alseth, Michael A. Brockhurst, Sam P. Brown, Edze R. Westra

**Affiliations:** 1 Environment and Sustainability Institute, Biosciences, University of Exeter, Penryn, United Kingdom; 2 Instituto de Biologia Molecular e Celular (IBMC), Universidade do Porto, Porto, Portugal; 3 Instituto de Investigação e Inovação em Saúde (i3S), Universidade do Porto, Porto, Portugal; 4 Center for Microbial Dynamics and Infection, Georgia Institute of Technology, Atlanta, Georgia, United States of America; 5 School of Biological Sciences, Georgia Institute of Technology, Atlanta, Georgia, United States of America; 6 Centre for New Antibacterial Strategies & Microbial Pharmacology and Population Biology Research Group, Department of Pharmacy, UiT—The Arctic University of Norway, Tromsø, Norway; 7 Division of Evolution, Infection and Genomics, School of Biological Sciences, University of Manchester, Manchester, United Kingdom

## Abstract

Attacks from molecular parasites such as mobile genetic elements (MGEs) have driven the evolution of defense systems in bacterial genomes. Yet, despite significant advances in understanding the molecular mechanisms of these bacterial immune systems, we have only a rudimentary understanding of their ecology and evolution. Bacteria exist as part of complex microbiomes, but community ecology and microbiome research has yet to characterize the impacts of interactions between MGEs and defense mechanisms upon the structure, dynamics and evolution of microbiomes. This Essay introduces and discusses the interplay between bacterial community dynamics and bacterial immune systems, speculating about how these reciprocal interactions may shape microbial community structure and function.

## Abstract 2

Bacterial immune systems have evolved in response to diverse molecular "parasites", yet their ecological roles remain poorly understood. This Essay explores how interactions between mobile genetic elements and bacterial defences shape microbiome structure and function.

## Introduction

Microbial communities are essential components of Earth’s ecosystems, having important roles in nutrient cycling, disease control, climate change regulation and environmental stability [1–3]. These communities are the invisible force behind essential biogeochemical cycles, influencing greenhouse gas emissions, carbon cycling, nitrogen fixation, and methane production. Disruptions to microbial functions can therefore have downstream effects on ecosystem stability and on planetary health [3].

Microbial communities are in turn governed by fundamental and overlapping ecological and evolutionary forces that can be summarized as selection, drift, dispersal, and diversification [4]. Interest in the structure, dynamics, and functions of microbial communities has been turbocharged over the past few decades by the advent of microbiome research, leading to an unprecedented trove of information on what microbes are where, and how microbiome features correlate with measures of human or environmental health [5,6]. A major challenge for the microbiome field is to move beyond descriptive and associative approaches and to establish bottom-up mechanistic understanding of the forces shaping the dynamics and functions of polymicrobial communities [7].

**Fig 1:**
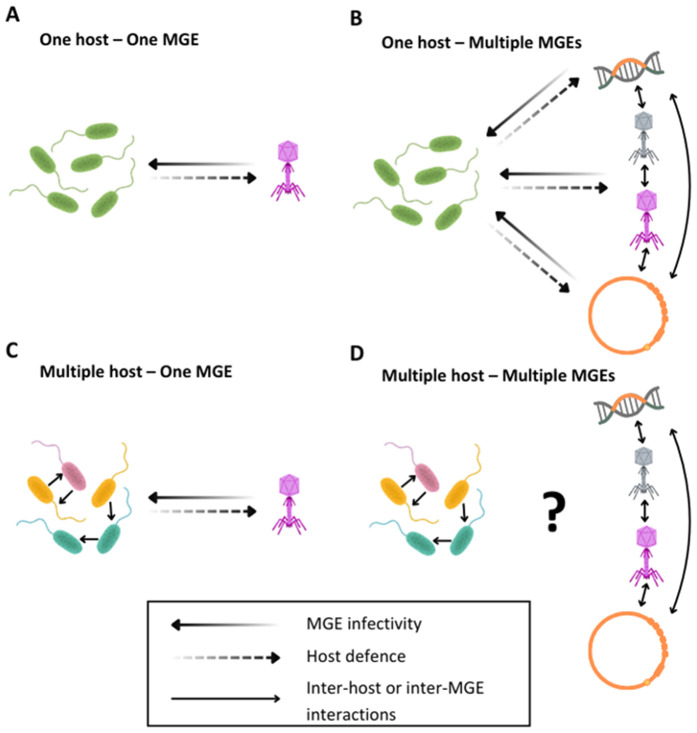
Moving beyond the one host–one parasite paradigm. **A.** The one host–one mobile genetic element (MGE) paradigm forms a critical basis for most work on molecular mechanisms, by isolating precisely defined interactions. It is also the current basis for most ecology–evolution and co-evolutionary studies. **B.** The one host–multiple MGEs paradigm is mainly used for fundamental work on potential applications for phage in biotechnology and/or medicine (e.g., phage cocktails for antimicrobial therapy). **C.** The multi-host–one MGE paradigm is used for ecology–evolution studies on competition in bacterial communities in which one species is targeted by a phage. **D.** The multi-host–multiple MGEs paradigm. The move from A (most MGE/defense work) to D (the real world) will allow us to ask community-level questions. Figure created in BioRender. Moura Alves, P. (2025) https://BioRender.com/v1ritd7.

We propose that a critical step towards building a mechanistic understanding of microbial communities is the full integration of mobile genetic elements (MGEs; Box 1) and MGE defenses into studies of microbial community structure and functioning. Microbial communities are teeming with diverse MGEs that can move horizontally between bacterial strains and species [8]. Some of these, such as lytic bacteriophages (phage; Box 1), are thought to impact microbial community composition [9–11]. Others, such as plasmids, can fuel adaptation through horizontal transfer of beneficial traits between bacterial species [12], while also imposing variable survival and fecundity costs [13]. Recently, hundreds of defense mechanisms that protect against detrimental MGE infections (also referred to as bacterial immune systems) have been discovered in bacterial genomes [14,15]. While much progress has been made in understanding the molecular mechanisms by which bacterial immune systems defend against MGEs within cells, we lack a fundamental understanding of how MGEs and the bacterial immune system interact in a microbial community context. Because MGEs have important and widespread effects on microbial community structure, dynamics, and function (Box 1), it is probable that MGE interactions with bacterial immune systems will, by extension, be important drivers of these processes. This gap in understanding arises from the fact that most experimental work to date has been focused on pairwise one host–one MGE model systems (Fig 1), and the challenges of extrapolating these simplified interactions to real world scenarios where bacteria exist in multi-species communities [16].

**Box 1. Glossary.**

**Abortive infection**

A host-controlled process leading to dormancy or cell death of infected bacteria before the phage can finish its replication cycle, preventing release of virions and onward infections. This response results from a wide range of defense mechanisms that detect infection and induce cell death, and is broadly distributed in bacterial genomes.

**Auxiliary metabolic genes**

Metabolic genes, often delivered by phages, that modulate or enhance host metabolism during infection to benefit the phage’s replication and survival.

**CRISPR-Cas systems**

Adaptive immune systems that utilize a genetic memory encoded by the CRISPR array to provide sequence-specific immunity against mobile genetic element (MGE) infections.

**Helper MGE**

A MGE that provides the necessary machinery (e.g., proteins involved in DNA transfer, replication, or integration) required for the movement or activity of another MGE that cannot mobilize itself independently.

**Hitcher MGE**

An MGE that relies on the mobility or replication functions of another MGE for its movement between bacterial cells.

**Horizontal gene transfer**

The transfer of genetic material between genomes, including both within and between species, and one of the most important driving factors of genetic diversity and evolution in bacteria.

**Lysogeny**

Part of the temperate phage life-cycle that involves integration of the phage’s genetic material into the host bacterium’s genome as a prophage or its maintenance as a circular replicon within the cytoplasm, allowing for the vertical transmission of the phage from a mother cell to a daughter cell.

**Lytic phage**

A phage with a life-cycle that hijacks the host cellular machinery to replicate and produce new virions that are released through lysis of the bacterial cell and can go on to infect new bacterial cells.

**Microbial community dynamics**

Temporal changes in community structure and function, driven by ecological and evolutionary forces acting on the community (e.g., selection, drift, dispersal, and diversification).

**Microbial community function**

The collective biochemical and ecological activities performed by a microbial community which contribute to ecosystem or host-level outcomes.

**Microbial community structure**

The identity, abundance, and spatial organization of microbial taxa (including MGEs) within a defined location and point in time, often summarized by diversity metrics and interaction networks.

**Mobile genetic elements**

Mobile segments of DNA that can move within or between genomes, including self-mobilizable elements, such as conjugative plasmids, and satellites and mobilizable elements that hijack the mobility machinery of other mobile elements.

**Phages**

Viruses that only infect bacteria and are a subtype of MGEs, due to their ability to mobilize genetic material between the host cells.

**Phage-inducible chromosomal island-like elements**

A family of highly MGEs that hitchhike on phages and spread between bacterial populations, contributing to host cells adaption, evolution, and virulence.

**Prophage**

A latent phage that is usually integrated into the host bacterial chromosome but may alternatively persist as a circular extrachromosomal cytoplasmic replicon.

**Restriction–modification systems**

A defense mechanism that uses methylation patterns to distinguish self from non-self. Restriction–modification systems encode a restriction enzyme that degrades invading mobile DNA.

We propose that tractable synthetic microbial communities, where multiple hosts and/or multiple MGEs coexist in controlled lab environments, can advance understanding of the role of bacterial immune systems in shaping microbial community features, and vice versa, with relevance to natural, agricultural, and clinical settings. In this Essay, we highlight how insights from various branches of ecology (including infectious disease ecology, community ecology, and evolutionary ecology) can help progress the field of bacterial immune system research into a multi-host, multi-parasite world.

## Bacterial immune systems and microbial communities

**Fig 2:**
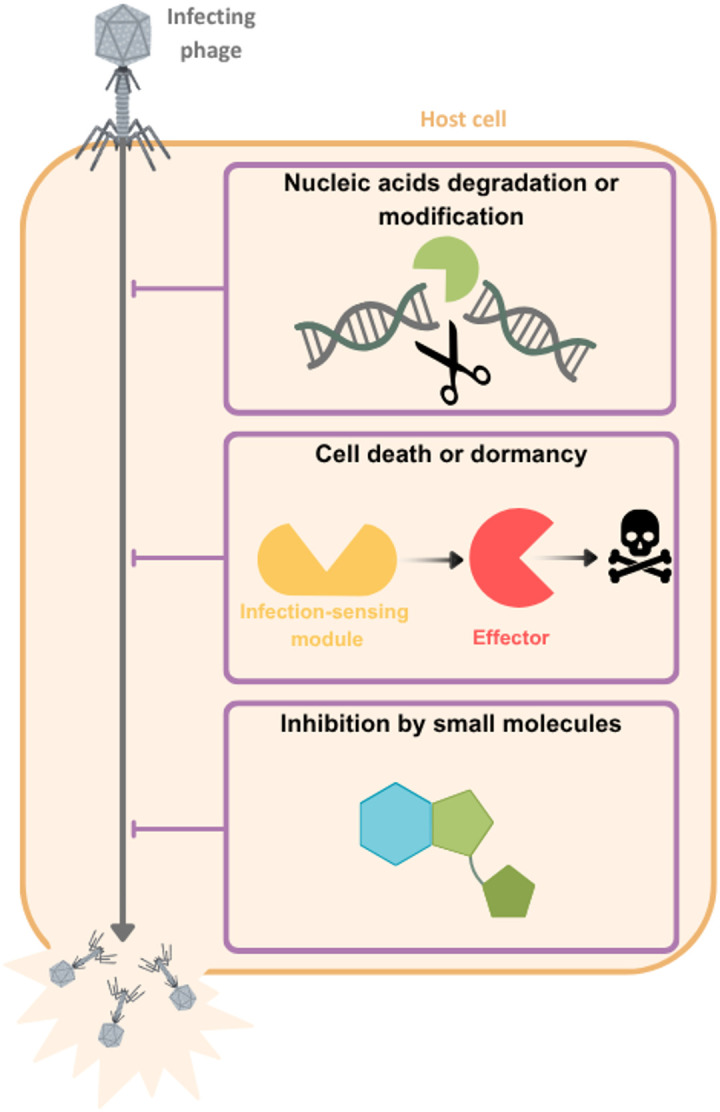
Overview of the main types of bacterial immune system mechanisms. The main modes of action of bacterial immune systems are shown. These strategies reflect distinct layers of bacterial immunity, ranging from targeted DNA cleavage or modification to altruistic cell death and phage inhibition by small inhibitory molecules that prevent replication. Figure created with Canva (www.canva.com).

The presence of parasitic MGEs (such as lytic phages) has selected for bacterial immune mechanisms that provide protection against MGE infection [17,18]. In metagenomic analyses, environments vary in the prevalence of bacterial immune systems, with gut communities enriched for immune systems compared with soil, ocean, or plant-associated communities [19,20]. Generally, environments with higher phage abundances also tend to be enriched for bacterial immune systems [21,22]. These patterns suggest that ongoing conflict between bacteria and parasitic MGEs selects for varied mechanisms for defense (Fig 2). The most common defense strategies are to cleave MGE genomes or transcripts (e.g., restriction–modification (RM) systems or CRISPR-Cas; Box 1) or to induce host cell death or dormancy (e.g., CBASS or Thoeris), but additional anti-MGE strategies have been uncovered recently [14,15,17]. In the past couple of years, more than 150 distinct bacterial immune system families have been identified and characterized [14]. Based on our current knowledge of the diversity of immune systems and the available sequenced genomes, it has been estimated that, on average, bacterial genomes encode around 5–6 immune systems [23]. The coexistence of multiple immune mechanisms in a single bacterium enables a multi-layered response to phage infection and/or a broader resistance range against a wider diversity of MGEs [17]. To date, the field has mostly focused on developing a mechanistic understanding of how these immune systems work in isolation, leaving ecological and evolutionary questions largely unanswered. In this section, we focus on the less explored questions of how bacterial immune systems may shape the structure and function of microbial communities, and how community composition may select for particular immune mechanisms.

### Effects of bacterial immune systems on microbial community structure and function

#### Direct effects of bacterial immune systems on MGE communities.

Bacterial immune systems per definition provide protection against MGE infections, and all else being equal, their presence should reduce MGE loads in a microbial community. However, very little is known about how the diversity of bacterial immune systems in a community shapes the MGE community. Some immune mechanisms are highly specific and may be able to distinguish between “good” (MGEs that provide fitness advantage) and “bad” (costly or harmful to the host) MGE infections, which could have major consequences for MGE community composition. For example, many bacterial immune systems recognize specific molecular patterns associated with phage infection [24], whereas others, such as Wadjet systems, specifically target plasmids [25]. Because of their specificity, these bacterial immune systems may suppress parasitic MGEs in the community while allowing beneficial MGEs to persist. Other defenses, such as type I-III RM systems, methylate self-DNA and restrict any unmethylated DNA that infects the cell [26], leading to broader resistance ranges and, potentially, to the suppression of a broader diversity of MGEs in the community.

Teasing apart how bacterial immune systems shape the MGE community is complicated by the fact that immune systems are often imperfect barriers to MGE infection. For example, bacteria with CRISPR immunity can still take up targeted plasmids, which can persist in the population if they are under positive selection [27]. Moreover, MGEs may evolve to overcome bacterial immune systems [18,28]. For example, it is now clear that many MGEs carry anti-defense genes that can block bacterial immune systems [18,29,30]. Because anti-defense genes are highly diverse in their sequences and mechanisms, it is expected that current annotations significantly underestimate the true prevalence of MGE-encoded anti-defenses [29]. Indeed, recent studies have revealed that anti-defense strategies are common in many conjugative plasmids, often encoded within the leading regions of the plasmid (which enter recipients first) to inactivate common bacterial immune systems immediately following infection [31,32]. Furthermore, another factor that complicates our ability to predict how defenses shape MGE communities is epistasis between different bacterial immune mechanisms. So far, phenotypic characterization of bacterial immune systems has mostly focused on individual mechanisms, but several studies have shown that different immune mechanisms can provide additive or synergistic levels of immunity when they co-exist in the same cell [33–36]. Hence, MGE spread in a community will likely depend not just on the complement of bacterial immune systems in the community as a whole, but also on their co-occurrences.

Finally, the activity of many bacterial immune systems and anti-defenses will be sensitive to environmental conditions. For example, CRISPR-Cas immune systems are affected by temperature [37] and bacteriostatic antibiotics [38], and anti-CRISPR activity can be impaired by sub-minimum inhibitory concentration levels of antibiotics that act on translation [39]. Thus, while it intuitively makes sense that the presence of specific defense mechanisms may directly influence the flux of MGEs in a community, to what extent they shape the MGE community composition remains an important open question. Teasing this apart requires more data on the strength and range of resistance associated with individual immune mechanisms and their combinations, as well as a deeper understanding of the host ranges and transfer rates of MGEs and their anti-defense strategies, as well as how each of these variables depends on environmental parameters.

#### Indirect effects of bacterial immune systems on broader community structure.

Bacterial immune systems can shape the broader community composition via indirect effects; for instance, by modifying phage-mediated “top-down control” of community structure through density-dependent predation. For example, in microbial communities, phages can exert effects on bacterial populations by preying on defenseless bacteria and therefore affecting the overall ecosystem stability [40].

Although in vitro experiments with simple communities (often 2 species) and theoretical models suggest phage to be important in shaping community structure, evidence for phage control in natural complex communities is mixed [40]. The recently uncovered diversity of bacterial immune systems in microbial communities and their ability to move between strains and species (discussed below) may dampen top-down control by phage in natural environments [41]. The reason for this may be partly explained by the “pan-immunity” hypothesis, in which diverse immune systems can be simultaneously present or shared among various bacterial strains within a community, contributing to a robust overall immunity against phage infection [42]. Such a system would consequentially foster an environment in which direct phage-induced mortality may be less impactful than in the simplified community models tested in vitro, particularly those of the one host–one parasite kind (Fig 1A).

Apart from shaping top-down control, bacterial immune systems may also have implications for bottom-up control (i.e., effects of resources on coevolutionary arms race and selection within the community structure). Phage and other MGEs can also encode auxiliary metabolic genes (Box 1) that may extend host metabolic functions during infection (e.g., during lysogeny; Box 1) [43]. In this context, defenses, through their interactions with MGEs, can contribute to bottom-up control of the microbial community composition. To our knowledge there are currently no studies that have investigated whether bacterial immune systems shape microbial community structure by modifying bottom-up control.

#### Bacterial immune systems partition gene flow in communities.

Horizontal gene transfer (HGT; Box 1) enables bacteria to exchange genetic material via three major pathways: transduction (transfer of DNA by phages); transformation (uptake of free DNA from the environment); and conjugation (transfer of genetic material through direct cell-to-cell contact via pili). Bacterial immune systems commonly restrict the acquisition of novel genes by HGT. Bioinformatic analyses support this idea for at least some defenses, such as CRISPR-Cas and RM systems [44–48], although this will depend on the bacterial species and the immune system sub-type [49]. Interestingly, some immune mechanisms may control gene flow in more sophisticated ways. For example, RM systems, which are found in approximately 90% of bacterial genomes, can partition gene flow within a microbial community. While HGT is reduced between strains carrying distinct (incompatible) RM systems, HGT is permitted between strains carrying similar RM systems, such that gene flow is determined by RM system compatibility rather than by phylogenetic similarity [50]. By contrast, experimental studies have shown that CRISPR-Cas immune systems can promote HGT, because of their sequence specificity. In *Pectobacterium atrosepticum*, CRISPR-Cas enhanced transduction by allowing the uptake of bacterial DNA while selectively targeting phage DNA [51], thus enabling HGT while also limiting the risk of phage infection. While these studies highlight important principles of the interactions between immune systems and HGT, observational and experimental research is needed with natural and synthetic microbial communities to tease apart how bacterial immune systems interact to shape HGT in more realistic multi-host–multi-MGE scenarios.

#### Impact of immunity on population and community functioning.

MGEs can accelerate the spread of novel functional traits such as antibiotic resistance, driving population- and community-level functional innovation [52]. Indeed, bacterial communities can accelerate evolutionary responses to novel challenges by aligning MGE fitness effects across host species [53]. In this context, immune systems can potentially limit the evolvability of their bacterial hosts by removing them from this collective process of adaptation. Consistent with bacterial immune systems being a potential barrier to innovation, a number of studies have shown that strong pressure for evolutionary innovation (e.g., drug exposure in pathogens) can select for the loss of CRISPR function and increased acquisition of drug-resistance MGEs [54–56].

The evolution of defense mechanisms themselves can also directly impact population and community function. This is particularly the case with surface factor modifications, which can provide protection against phages by limiting phage binding, but at same time can eliminate functions that are mediated by the surface receptor. The trade-off between defensive benefits and functional costs is modified in a community context, with surface factor modifications less frequently observed in a community setting, and CRISPR-Cas more common [57]. The increasing use of CRISPR-Cas allows for the maintenance of surface-factor-mediated functions in the community.

Although some experiments show that lytic phage can influence microbial community composition and stability, the type of defense evolved has no lasting impact on community dynamics under simple laboratory conditions [9]. For example, a study using a four-species bacterial community containing either wild-type *Pseudomonas aeruginosa* or a CRISPR-deficient mutant plus a lytic phage demonstrated that, over a 10-day evolution experiment, the presence of functional CRISPR-Cas immune system clones did not alter the community structure when compared to *P. aeruginosa* that evolved phage-resistance via surface factor modifications [9]. Similar results have been observed in experiments on plant microbial communities, where the presence of phages affect the most common or fastest growing bacteria following kill-the-winner dynamics, consequently affecting community composition over time [58]. That said, most work to date either involves a simplified set-up with little defense or MGE diversity [9], or does not look closer at the potential impact of defense mechanisms [58], leaving us to wonder how outcomes may change when these considerations are taken into account. This point is of great pertinence, as bacteria need to use their arsenal of defenses to constantly distinguish ‘good’ from ‘bad’ MGE infections, which in turn will likely have community-wide effects.

### Effects of microbial community structure on bacterial defenses

#### Community interactions can drive exposure to novel MGEs and therefore selection for new defenses.

Life in a community introduces the risk of parasite “spillover” or “host jump” events that introduce novel parasites into a focal population. In a human context, these events are termed “zoonoses” and are the subject of a broad literature [59,60] that offers potential insights into MGE dynamics in a community context.

Longstanding work in human infectious disease ecology points to several ways that communities can promote novel infection acquisition in a focal species, particularly and paradoxically in the context of community biodiversity loss [60]. Habitat degradation can promote the abundance of a smaller number of generalist species (e.g., bats or rodents) that disproportionately carry potential human zoonotic diseases [60]. Interestingly, this can be likened to the use of antibiotics, which disrupts the bacterial community via the selective pressure that favors bacteria harboring MGEs [61], resulting in the enrichment and spread of antibiotic resistance genes and putative immune systems. Processes of host species movement and migration can also increase novel pathogen acquisition by increasing encounter rates with new parasites. Migrating individuals can have substantial impacts on resident communities when residents lack appropriate defenses to novel parasites; for example, the introduction of American gray squirrels into the United Kingdom almost entirely eliminated resident red squirrels due to their lack of defense against an imported virus that was tolerated by the gray squirrels [62]. This process of parasite-mediated competition is an example of the ecological principle of ‘apparent competition’ [63].

The red/gray squirrel example of apparent competition has parallels at a microbial community level, notably in the case of competition mediated by temperate phage [64–66]. Carriers of the phage (lysogens with chromosomally-integrated prophage; Box 1) have the ability to “weaponize” their domesticated phage, as their lysogenic kin are immune to the weapon due to expression of the phage repressor gene, while competitors are potentially susceptible [64,67]. Altogether, these dynamics underscore that, as in macroecological systems, the structure, diversity, and connectivity of microbial communities critically shape the risks and outcomes of parasite transmission and evolution.

#### Community context can limit exposure to MGEs.

While life in a community can introduce the possibility of novel parasite exposure, it can also limit the overall rate of parasite exposure via a range of ecological processes [60]. Communities can suppress the density of a focal species, which can limit the ability of a host-specialist parasite to persist in the focal species population (given density-dependent transmission). In the context of more generalist parasites that can move between species, community diversity can also limit parasite exposure via processes termed “dilution effects” [60]. Dilution effects occur when diverse communities limit host transmission due to the increased presence of non-competent hosts. Lyme disease provides a human example, in which forest diversity can reduce the rate of human infection by diverting ticks to less efficient hosts [68]. In bacteria, the dilution effect occurs when the presence of non-infected or less-infected bacterial populations reduces the overall transmission potential of MGEs, thereby limiting their spread within a community [69]. This phenomenon is particularly relevant in environments where diverse bacterial populations coexist, as it can hinder the establishment of MGEs by decreasing the likelihood of successful HGT events. The examples in this section and the previous section draw heavily on the human zoonosis literature. We note that this literature contains an exciting set of concepts and hypotheses that could be directly tested using microbial systems. Whether community contexts enhance (see previous section) or limit exposure to MGEs will in turn shape the strength of selection on mechanisms of MGE defense.

#### Community context can shift the balance of defenses via shifting costs and benefits.

Many studies have established that bacterial immune systems can come with trade-offs; although they can help to prevent or mitigate the damaging effects associated with MGE infections, they also carry fitness costs that are frequently caused by autoimmunity, which reduces bacterial fitness in the absence of infection [57,70–72]. Recent work has shown that increased expression of defense mechanisms can broaden the protection range of bacteria while also increasing the risk of autoimmunity [73]. How these costs and benefits of immune systems depend on the presence or absence of a wider microbial community of bacteria, and the interaction types between these bacteria, has rarely been explored.

In one study [57], competition between *P. aeruginosa* and *Acinetobacter baumannii* in the presence of *Pseudomonas* phage DMS3vir resulted in amplified fitness costs of receptor mutations that conferred phage resistance to *P. aeruginosa*. By contrast, the cost of CRISPR immunity was not affected, hence resulting in stronger selection for CRISPR-Cas immune systems in a community context, although this benefit was largely lost over time as the phage is driven extinct [57]. A broader understanding of why MGE infections and bacterial immune systems become more costly during interspecific competition, why this varies across competitor species, and how it varies across environments will be required to predict and manipulate MGE–immune interactions in more natural contexts.

The cost–benefit of specific bacterial immune systems can also be modulated by the spatial structure of bacterial communities, as spatial structure is predicted to change the costs and benefits of defenses that feature individual cell suicide [70]. Consider the case of abortive infection (Abi) systems (Box 1). Programmed cell death (following infection) can provide benefits to neighboring cells by protecting them from infection. In a spatially structured population, those neighboring cells are likely to carry copies of the same Abi genes, therefore providing a kin-selected benefit to the self-sacrificing cell. By contrast, in a well-mixed population, the neighboring cells are no more likely to carry the same genes as any other individuals in the population, and therefore selection for this cooperative sacrificial trait is not maintained, leading to a predicted loss of Abi or similar group-protective mechanisms [70].

MGEs such as plasmids, prophages, and integrative elements are often considered to be parasitic (exploiting host resources for replication), yet MGEs may adopt a dynamic position along a continuum from parasitism to mutualism, depending on environmental and ecological conditions [74,75]. Accordingly, bacterial defense mechanisms, such as CRISPR-Cas and RM systems, are no longer viewed solely as exclusion mechanisms but as adaptive regulators capable of modulating MGE “infections” in a context-dependent manner [47,76]. For instance, in stressful environments, MGEs are more likely to carry useful genes, including those encoding antibiotic resistance, toxins, or metabolic enzymes [13,75], reversing the cost–benefit equation on defenses against MGEs. Hence, host immunity systems may filter their activity in order to balance genome protection with the acquisition and retention of advantageous elements [77]. For example, in *Enterococcus faecalis*, although CRISPR-Cas systems typically restrict MGE uptake and antibiotic resistance, it was recently shown that, under antibiotic selection, CRISPR-Cas activity becomes compromised, enabling plasmid persistence and enhanced HGT, whereas in the absence of selection, plasmids are lost unless CRISPR-Cas is absent [78,79]. Overall, the ecological context likely has a critical role in defining the relationship between MGEs and the host immune system. What is parasitic under one condition may become mutualistic under another, and bacterial immune systems seem to have evolved mechanisms to sense and respond to these changes. This suggests a more nuanced view of bacterial immune system–MGE interactions in which, rather than a static conflict, these relationships are part of a dynamic evolutionary and ecological framework.

#### Community context can allow distributed immunity and enhanced protection.

Although individual immune mechanisms may be more costly in a community context, the diversity of defense strategies across bacterial strains and species may enhance the overall defensive capabilities of the community. Analogous to the diversity of CRISPR spacers that naturally evolves from an initially clonal population in response to phage to provide robust population-level defense [80], the presence of diverse immune systems across bacterial species may create a more robust protective environment against MGE infections. This effect is known as “distributed immunity”, and suggests that the collective immune repertoire of a bacterial population is more effective than that of individual strains [81]. Recent metagenomics studies show that there is a huge diversity of bacterial immune systems contained within individual communities [19]. To what extent the efficacy of each individual bacterial immune systems is shaped by the community structure and the genetic diversity present within it remains largely unknown.

Moreover, because bacterial immune systems are often mobile [25,49,82], HGT can be utilized to share access to defense mechanisms. This “pan-immune system” hypothesis [42] suggests that the “effective” bacterial immune system is defined by the pan-genome of those bacteria able to share genetic resources, and not just the defenses encoded by one single cell [42]. According to this model, microbial species can rapidly adapt to infection by MGEs through the sharing of defenses, with defense diversity and dissemination enhancing the levels of distributed immunity [42]. Consistent with the pan-immune system model, research on *Vibrio cholerae* has demonstrated rapid horizontal transfer of defense mechanisms in microbial populations, often as a consequence of phage infection [83]. Yet, one cannot exclude that pan-immunity may be partly driven by the selfish nature of MGEs, which often carry their own immune systems. This provides a fitness advantage to the host, while ensuring the propagation of their own genes, thus contributing to the spread of defense mechanisms (sharing of public goods) across the population in a non-altruistic fashion.

Alternatively, the community structure may promote protection via the super-spreader effect, which suggests that certain bacterial strains can act as reservoirs of MGEs and further facilitate their transmission to other strains. This increased genetic mobility driven by super-spreaders may enhance the bacterial community fitness as a whole [84] while also raising questions about whether these super-spreaders encode fewer immune systems and/or influence the selection of specific defenses in neighboring cells.

If and how immune systems are shared between strains and species in a community will depend on the recipient strain as well as the structure of the wider microbial community, its genetic features, the ecological conditions, and the species–species interactions in the community [19,85]. Moreover, as the ability to exchange genes depends on immune systems in the donor and recipient species (see above), exchange of both immune systems and MGEs may lead to the emergence of genetic associations that are independent of phylogeny. Teasing apart the consequences of mobility of both bacterial immune systems and MGEs on the patterns of HGT and genetic associations will benefit from formalizing the pan-immune model into a predictive framework that yields testable predictions [85,86].

## Defense mechanisms in microbial communities function beyond host protection

Classically, immune systems have been considered to protect bacterial genomes against MGEs. However, recent research has revealed that numerous immune mechanisms are encoded within MGEs themselves [25,49,82]. Given that many defenses themselves are encoded on MGEs [82,87–90] and function beyond their role of protecting the host cell they reside in, this raises questions of “agency,” with defense mechanisms serving as a means to protect their host MGE (that also resides within a bacterial host cell, like a genomic Matryoshka doll) from potential competitor MGEs [47]. Immune systems encoded by MGEs may shape MGE–MGE interactions, which has potential knock-on effects for microbial community structure and function that remains to be explored.

**Fig 3:**
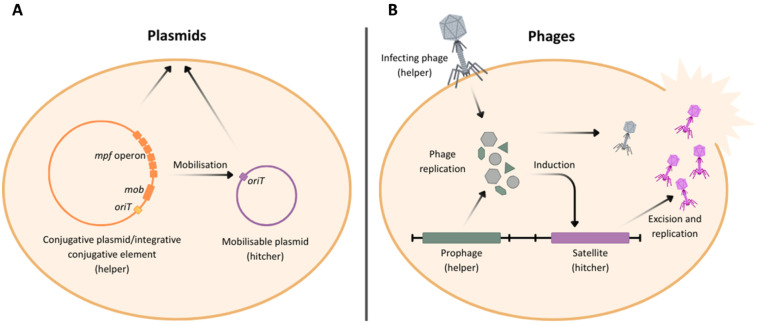
Helper–hitcher relationships among mobile genetic elements. These interactions illustrate how non-autonomous mobile genetic elements co-opt the transfer mechanisms of autonomous elements to facilitate their own dissemination. **A**. In plasmid systems, conjugative plasmids or integrative conjugative elements act as “helpers” by encoding the mating pair formation (*mpf*) operon and mobilization (*mob*) functions, enabling the transfer of mobilizable plasmids (“hitchers”) that carry only an origin of transfer (*oriT*). **B**. In phage systems, an infecting or resident “helper” phage can induce the activation of a prophage and support replication. Satellite phages (“hitchers”) exploit the structural and replication machinery of the helper phage to undergo excision, replication, and packaging. Figure created with Canva (www.canva.com).

For example, many MGEs can be exploited by so-called “hitchers,” which use the “helper” MGE to mobilize (Fig 3; Box 1). Hitcher MGEs cannot independently transfer between genomes and fully rely on helper MGEs to do so [91]. Hitchers benefit, as they may reach a broader range of hosts [91–93], but this interaction can be costly for the helper MGE. For example, the satellite phage-inducible chromosomal island-like elements (PLEs; Box 1) of *V. cholerae* are hitcher elements that exploit the lytic phage ICP1 to move between *V. cholerae* genomes [83]. As PLEs are unable to perform their own replication, they redirect the ICP1 machinery. Most cells will instead undergo abortive infection, with sporadic release of transducing PLE particles, which are smaller than the ones produced by ICP1 and do not support packaging of the phage genome. As a result of these interactions, PLE provides highly effective resistance to ICP1 infections, and this in turn has exerted strong selection for ICP1-encoded nucleases that cleave the PLE element. Approximately half of all ICP1 isolates carry a Type I-F CRISPR-Cas system that cleaves the PLE element in a sequence-specific manner [83,94,95]. Some ICP1 phages that lack a CRISPR-Cas system encode an alternative endonuclease in the same locus called Odn, which also supports ICP1 replication in cells carrying PLEs [96]. In return, PLEs can overcome targeting by ICP1 through point mutations in the nuclease recognition sequences, leading to a coevolutionary arms race between these elements [95,97]. The presence of ICP1 in patient stool samples is negatively associated with the severity of disease [98], and evidence suggests that the acquisition of novel PLEs that provide resistance against ICP1 may underpin a selective sweep in one of the largest cholera epidemics in recent times [99].

Yet despite the importance of defense mechanisms in the battle between bacteria and MGEs, and consequently the spread of potentially “high risk” genetic material [100,101], we still know little of how universally true this is and what role it has in shaping microbial communities. In some hitcher–helper interactions, the fitness of helper phages seems to be unaffected by hitchers, with both MGEs creating copies during infection [91]. In at least some of these cases, immune systems are encoded by hitcher elements that benefit the helper element by targeting competitor MGEs [88,90]. Future research on MGE–MGE interactions may help to identify general rules on how MGE investment in defense mechanisms depends on the ecology and structure of microbial communities, and how this in turn shapes the structure and function of these communities.

## Conclusions and future perspectives

The intricate and dynamic interplay between microbiomes and bacterial immune systems is multifaceted, with microbial communities potentially influencing and being influenced by the evolution of bacterial immune mechanisms. Understanding microbiomes as both a driver and a consequence of bacterial defenses is essential for uncovering the ecological and evolutionary forces that shape microbial community structure and function. By combining insights from one host–one MGE systems and the ecological distribution and function of bacterial defense mechanisms, we can start to integrate that knowledge into multi-host–multi-MGE synthetic communities.

Several open questions (Table 1) remain that are intrinsically multi-scale, linking molecular mechanisms operating within cells to community ecological processes emerging at the level of dynamic microbiomes. Given recent technological and methodological advances that have significantly improved our ability to study microbial communities, now is the right moment to address those questions (Table 1). For instance, the latest metagenomic sequencing approaches could be used to link hosts with respective MGEs [102–107] or immune system repertoires [21,22] and shed light on the dynamics and mobility of genetic material within complex ecosystems. Mathematical modeling can provide insights into processes that are consistent with observed data [108], generate predictions that are testable experimentally [109] and test the logical validity of verbal hypotheses [110].

**Table 1:** Experimental strategies that can be applied to fill the knowledge gap between one host–one MGE systems and multi-host–multi-MGE ecological and evolutionary dynamics. We focus on experimental approaches to specific open questions, but underline that experimental strategies are complementary to theoretical and bioinformatic approaches to the same core questions. AI, artificial intelligence; MGE, mobile genetic element.

Open question	Experimental strategy	Objective	Ref.
Do bacterial immune systems limit or promote MGE diversity? And indirectly, bacterial diversity?	Factorial synthetic-community experiments	Defined microcosms allow testing of MGE–immunity–community structure dynamics	[57,111]
How does MGE infection shape community composition and immune system–MGE mobilization?	Time-shift assays in natural communities	Longitudinal sampling reveals temporal dynamics of MGE spread and host immune responses	[86,112]
	Single-cell mapping (sequencing and imaging)	Single-cell sequencing and spatial mapping directly link MGEs to their hosts	[113,114]
How much of the uncharacterized MGE cargo still encodes novel defense and counter-defense systems?	Metagenomic mining of ‘defense islands	Use of bioinformatic analysis to identify gene families that are enriched near known defense systems. Experimental validation by cloning putative systems into model organisms.	[25]
	AI structure-based prediction tools	Use of AI-based predictions of protein structure, interactions, and catalytic activity can generate numerous novel candidates	[115,116]
	Forward genetics approaches	Use of transposon library mutants can reveal distinct phenotypes compared to wild-type strains under MGE infections and subsequent identification of novel defense mechanisms	[34]
How does spatial structure affect MGE transmission and immune defense spread?	Spatial structure and microfluidic assays	Applying microfluidics and the study of structured communities can reveal how physical layouts modulate MGE–immune system dynamics	[117]
Do MGE–defense interactions provide new opportunities for microbiome management and manipulation?	Experimental evolution under different environments or stresses	Evolution experiments with defined MGEs and defense systems can reveal adaptive trade-offs in MGE–immune system interplay and highlight pathways for potential optimization	[118,119]
Are defense mechanisms identified in one host–one MGE representative of major mechanisms in multiple hosts–multiple MGEs?	Comparative genomics combined with shotgun metagenomics	Enables the identification of potential immune systems and comparison across environments spanning low- to high-microbial complexity	[21,22,120]
Do MGEs travel faster or slower through bacterial communities and how is transmission rate dependent on the arsenal and distribution of immune systems across a community?	Barcoded MGE tracking	Barcoding allows high-resolution tracking of MGE transmission events within a focal community	[113]
Can we predict MGE stability based on host–immune–environment interactions?	Combine modeling with targeted experiments	Theoretical models and experimental validation can reveal rules of MGE–immune system dynamics in bacterial communities	[109,121]
How do MGEs and defenses shape community-scale functions?	Functional studies (metaproteomics and metabolomics)	Use of omics to unravel compositional changes and functional shifts driven by bacterial immune system–MGE interactions	[122,123]

Crucially, these hypotheses can be further validated using synthetic microbial communities. By tailoring species composition, interaction types, defense mechanism profile, and MGE infections within a microcosm [9,57,124,111], one can manipulate the environment in order to experimentally validate theoretical predictions. Furthermore, single-cell microscopy techniques can be used to directly visualize MGE transmission events at cellular resolution. For example, recent imaging-based approaches integrating single-molecule DNA fluorescence in situ hybridization have been applied to map MGEs and their bacterial hosts, allowing the study of MGE distribution within microbiomes [113].

Overall, to address these questions will require a multidisciplinary approach, combining molecular insights with ecological and evolutionary principles, and the integration of experiment, bioinformatics, and theory (Table 1). These interdisciplinary efforts will provide crucial insights into microbial interactions, adaptation, and the broader evolutionary landscape of bacterial communities, and may help to manipulate the spread and function of clinically important genes, strains, and species within microbial communities.

## Abbreviations

AI: artificial intelligence
HGT: horizontal gene transfer
MGEs: mobile genetic elements
RM: restriction–modification
